# Streamlined Production of Recombinant Adeno-Associated Viruses Using Gateway Cloning Technology for Neural Circuit All-Optical Interrogation

**DOI:** 10.64898/2026.09.22.752944

**Published:** 2026-10-28

**Authors:** Annick Ayon, Abdelali Jalil, Coailinn Egan, Bertrand Ducos, Caroline Mailhes-Hamon, Astou Tangara, Benjamin Mathieu, Alexis-Pierre Bemelmans, Isabel Llano, Laurent Bourdieu, Stéphane Dieudonné, Vincent Villette, Jonathan Bradley

## Abstract

The advent of optogenetics and the rapid development of genetically encoded actuators and reporters for calcium, voltage, neurotransmitters, and other molecules have revolutionized neuroscience research by enabling precise, non-invasive optical interrogation of neural circuits. Successful implementation, however, critically depends on efficient reporter expression in defined neuronal populations. We present a Gateway®-based cloning platform providing a robust pipeline for rapid, efficient construction of recombinant adeno-associated virus (rAAV) vectors optimized for neuroscience applications. This platform addresses promoter selection, indicator engineering, and capsid-type optimization, and enables systematic optimization of vector components across a range of optical tools. The benefit of this modular approach is illustrated in the case of ULoVE, a two-photon microscopy method based on acousto-optic deflectors (AODs). This method provides serial light-targeting with kHz sampling rates and high signal-to-noise ratio in vivo, which imposes stringent requirements on indicator expression — including cell-type specificity, sparse labelling, and precise control of expression levels— efficiently met through the combinatorial flexibility of the Gateway pipeline. As specific examples, Gateway-constructed Cre-driver viruses combined with Cre-dependent reporters enabled cell-type-specific labelling for two complementary applications. In cerebellar Purkinje cells, an L7::Cre driver virus paired with a Gateway-constructed voltage indicator (JEDI2P-Kv) and ULoVE two-photon excitation at ∼5 kHz enables resolution of sub-millisecond dendritic voltage dynamics in awake, behaving mice, including optical detection of dendritic spikelets previously accessible only via intracellular electrophysiology. The same driver virus paired with calcium indicators (GCaMP6f, jRGECO1a) resolves climbing fiber-evoked calcium kinetics under the same conditions. In acute cerebellar slices, a kit::Cre driver virus enabled cell-type-specific expression of the optogenetic actuator ChR2(H134R) in molecular layer interneurons, where ULoVE doughnut-pattern photostimulation achieved single-cell, sub-millisecond optogenetic activation with micron-scale spatial resolution.

Beyond ULoVE applications, the same sparse, strong labelling also proved suitable for anatomical tracing of axonal projections in cleared cerebellar tissue, resolving individual deep cerebellar nuclei (DCN) axon terminals at the granule cell layer–molecular layer boundary. Taken together, our dual-front approach—combining a modular AAV expression pipeline with AOD-based optical acquisition—provides an integrated platform for developing sophisticated optical tools, including but not limited to AOD-based microscopy, for neural circuit interrogation.

## Full Text Availability

The license terms selected by the author(s) for this preprint version do not permit archiving in PMC. The full text is available from the preprint server.

