# Supplementary material for: Streamlined Production of Recombinant Adeno-Associated Viruses Using Gateway Cloning Technology for Neural Circuit All-Optical Interrogation
Source: bioRxiv. 2026 Sep 25:2026.09.22.752944. Preprint. [Version 1] doi: 10.64898/2026.09.22.752944 (PMC13622121; doi:10.64898/2026.09.22.752944)
Supplement: Supplementary file 1 [file media-2.pdf]

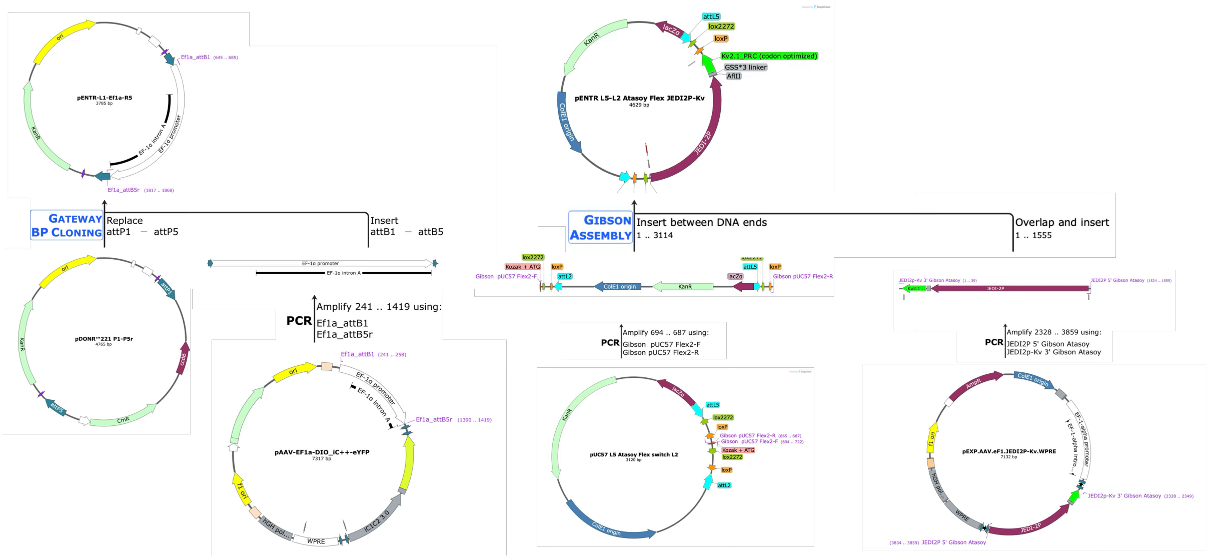

Supplement to Figure 2. Generation of pENTR plasmids by BP recombination and Gibson assembly.

(Left), BP recombination route. The sequence (or gene) of interest, here the eF1α promoter, is amplified by high-fidelity PCR using primers that append attB1 and attB2 sequences at the 5′ and 3′ ends, respectively (with incorporation of a Kozak consensus sequence upstream of the start codon if a coding sequence was being targeted). BP Clonase enzymes mediate recombination between the attB-flanked PCR product and the attP sites of a pDONR vector, producing a pENTR plasmid in which the eF1α promoter sequence is flanked by attL1 and attL2 recombination sites and the donor plasmid's ccdB cassette is simultaneously excised. Positive (KanR) and negative (ccdB toxicity in sensitive strains) selection ensure recovery of correct recombinants. The resulting pENTR plasmid serves as a permanent, sequence-verified repository for eF1α promoter use in subsequent LR reactions.

(Right), Gibson assembly route for conditional expression constructs. For GoI sequences intended for conditional (CRE- or FLPo-dependent) expression, sequences are inserted into a pENTR plasmid pre-configured carrying a recombination cassette of heterotypic pairs of either lox/Flex switch (Atasoy et al., 2008) or frt/fDiO (Fenno et al., 2014) recombination sites. In this case, the sequence of the genetically encoded voltage indicator JEDI2P-Kv is amplified with primers that incorporate overlapping sequences matching the insertion site within the recombination cassette, and Gibson assembly is used to insert the GoI between the recombinase recognition sites in the anti-sense orientation. The resulting pENTR plasmid carries JEDI2P-Kv flanked by attL recombination sites and internally flanked by heterotypic pairs of lox sites, such that CRE recombinase activity in the final transduced cell catalyses permanent reversion of JEDI2P-Kv to its functional sense orientation. As for the BP route, all pENTR clones generated by Gibson assembly undergo comprehensive Sanger sequence verification across the entire expression cassette before use. The example shown illustrates construction of pENTR-L5-Atasoy-Flex-JEDI2P-Kv-L2 (4620 bp), in which JEDI2P-Kv (1555 bp, with Kv2.1 soma-targeting motif) is inserted into the Flex switch cassette (Atasoy et al., 2008) of a pUC57-based backbone, producing a plasmid suitable for MultiSite LR recombination with a promoter entry clone and destination vector to generate CRE-dependent JEDI2P-Kv rAAV expression constructs.

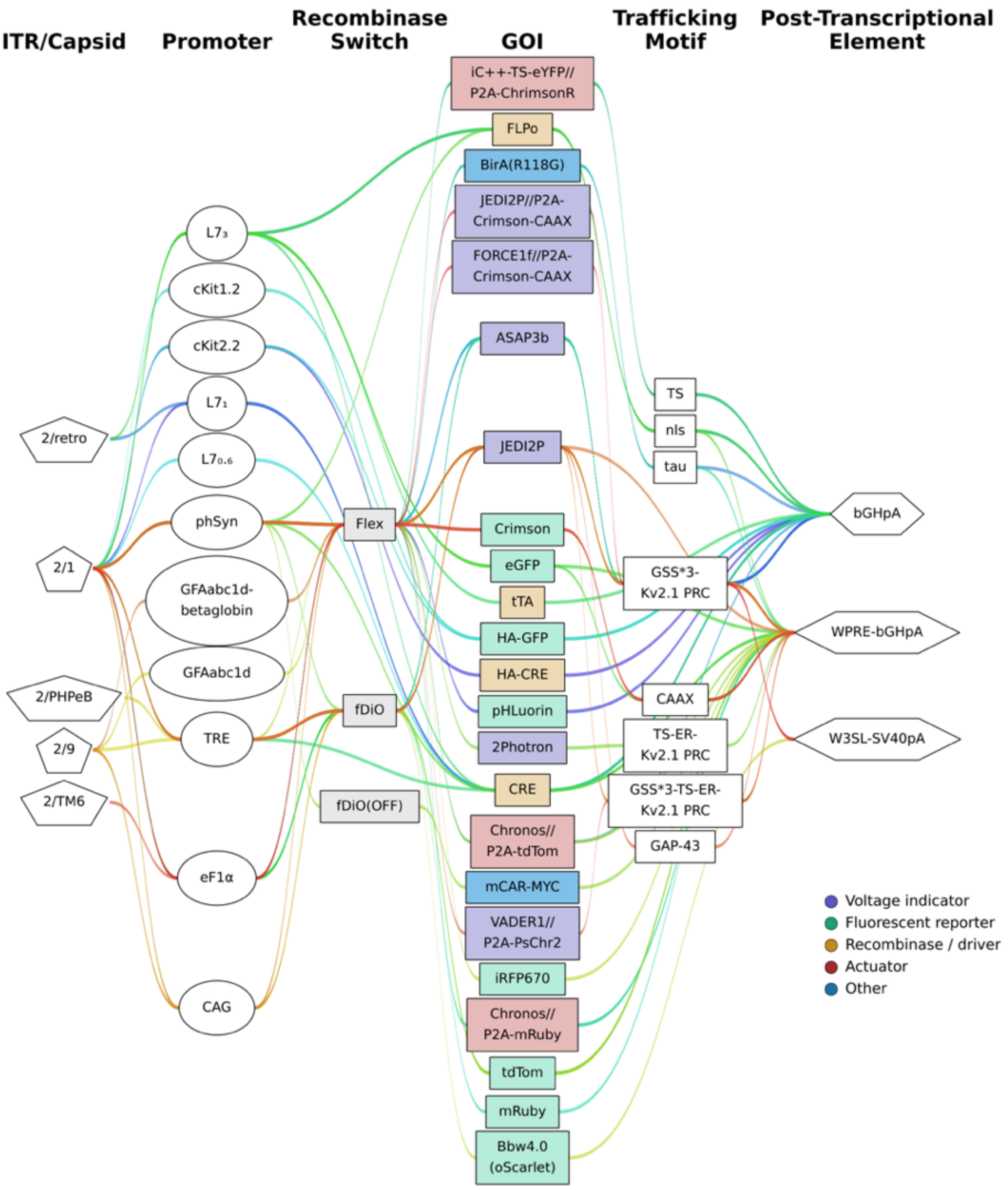

Supplement to Figure 3. Sankey diagram: Diversity of the 69 different rAAV expression constructs produced using the Gateway cloning pipeline.

Each colored line represents one Gateway construct produced with the cloning pipeline, tracing the component elements of that construct from left to right in canonical expression order: ITR/capsid types, promoter, conditional expression configuration, GoI (GEVI/reporter/recombinase or transactivator/actuator or other), subcellular targeting motif, and post-transcriptional regulatory elements.

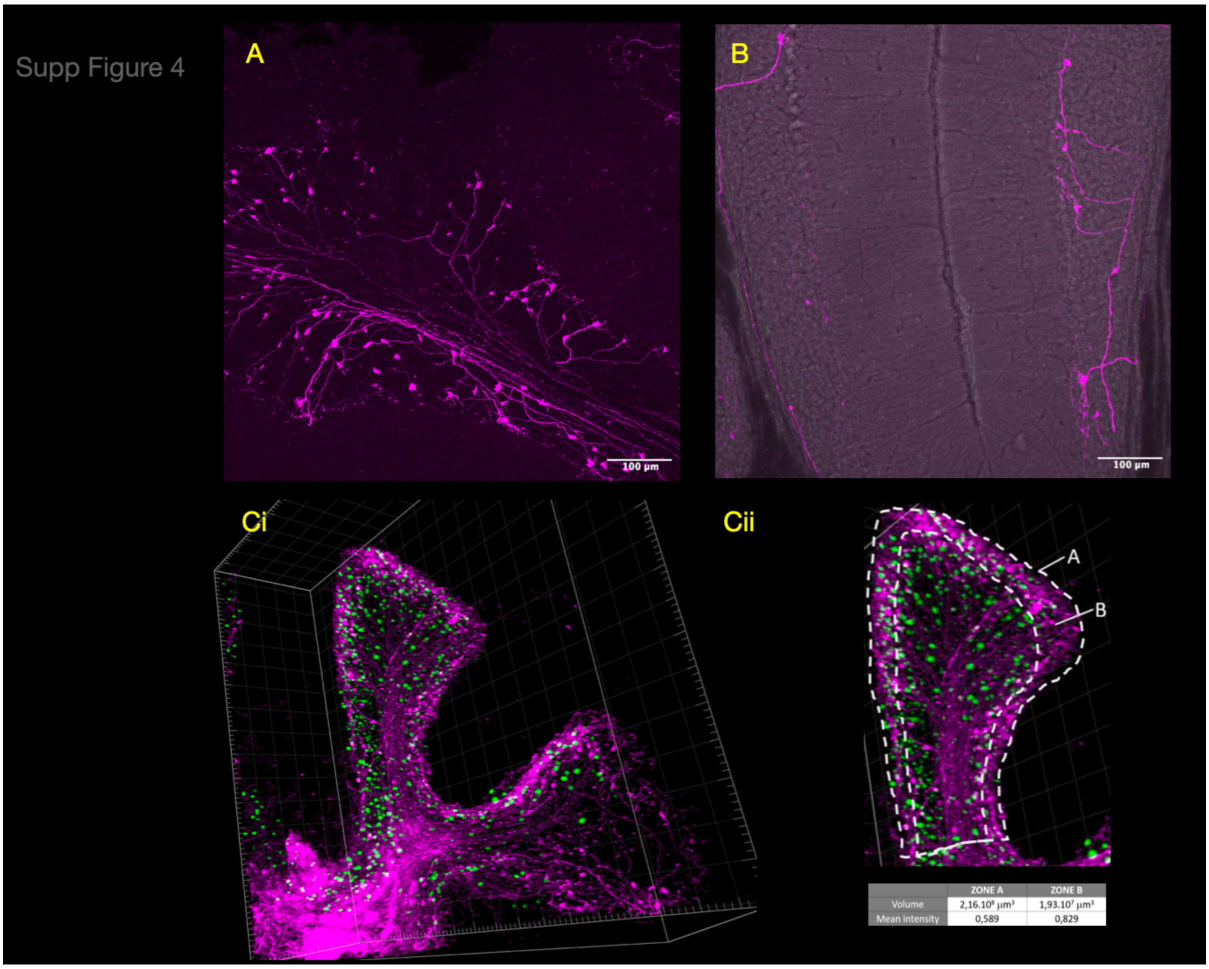

Supplement to Figure 4. Anterograde tracing of vGlut2+ DCN axonal projections to the cerebellar cortex. (A) Confocal image of a vibratome section showing tdTomato-labelled axons (magenta) from vGlut2+ DCN neurons, labelled using the triple-intersectional strategy described in **Figure 4**, with dense terminal boutons concentrated at the boundary between the granule cell layer and the molecular layer **(see Figure 5A for a schematic of the cerebellar cortical cytoarchitecture)**. Scale bar, 100 µm. **(B)** Confocal image from a separate section with sparser density of fibers, showing individual tdTomato-labelled vGlut2$^+$ DCN axons (magenta) and their terminal boutons at the granule cell layer–molecular layer boundary. Scale bar, 100 µm. **(Ci)** 3D reconstruction of a cerebellar lobe imaged in a 3 mm-thick sagittal slice, CUBIC-cleared and mounted in CUBIC R+ (Leica SP5, 10× XLPLN10XSVMP objective (NA = 0.6, WD = 8.0 mm, Olympus), showing tdTomato-labelled DCN axons and terminals (magenta) and GlyT2::eGFP-labelled glycinergic neurons (green, as in **Figure 4**). **(Cii)** Z-projection of the same reconstruction with two 3D zones outlined for quantitative comparison: Zone A, corresponding to the entire lobe excluding the molecular layer (where no axonal signal is present), and Zone B, corresponding to the Purkinje cell layer and superficial granular layer. Mean signal intensity in Zone B was 40% higher than in Zone A (Zone A: volume 2.16×10^8 µm^3, mean intensity 0.589; Zone B: volume 1.93×10^7 µm^3, mean intensity 0.829), consistent with the preferential targeting of DCN axon terminals to the granule cell layer–molecular layer boundary observed in **A** and **B**.
